# Harnessing Sex Reversion via Chemical Intervention in *Cannabis sativa* L.

**DOI:** 10.3390/plants15091291

**Published:** 2026-04-22

**Authors:** Lennard Garcia-de Heer, Tobias Kretzschmar, Jos Mieog

**Affiliations:** Faculty of Science and Engineering, Southern Cross University, 1 Military Road, Lismore, NSW 2480, Australia; lennard.garcia-de.heer@scu.edu.au (L.G.-d.H.); tobias.kretzschmar@scu.edu.au (T.K.)

**Keywords:** sex expression, plant hormones, plant growth regulators, dioecious, sex chromosomes, fertility, sex reversion, foliar spray, flower development

## Abstract

*Cannabis sativa* is a multipurpose dioecious species whose crop performance is governed by sex expression. Although sex is genetically determined by an X/Y chromosome system, plants can develop flowers of the opposite sex through sex reversion, commonly induced by manipulating endogenous hormone levels using plant growth regulators (PGRs). Here, we evaluated the effectiveness of PGRs that promote or inhibit major hormone pathways implicated in plant sex expression. Male and female clones from two accessions were treated with foliar applications of nine PGRs and four combinatory treatments to assess sex- and genotype-specific responses. Floral biomass and the proportion of each sex were recorded at harvest to assess treatment effectiveness. Ethylene emerged as the primary regulator of chemically modulated sex reversion in *C. sativa*, with its inhibition by silver thiosulfate inducing strong female-to-male reversion and its promotion by ethephon inducing equally strong male-to-female reversion in the inflorescences. Gibberellin promotion on its own resulted in female-to-male reversion at the axial nodes only, while its inhibition showed no reciprocal effects. The combination of silver thiosulfate and gibberellic acid resulted in the most complete female-to-male reversion, and all sex-reverted flowers were fertile. Together, the results indicated that flowers at axial nodes and at the terminal ends of inflorescences are under different hormonal control. Cytokinins, auxins, and jasmonates were found to exert minimal influence on sex reversion. All treatments exhibited pleiotropic effects, particularly gibberellic acid and paclobutrazol, which altered resource allocation, shifting biomass away from and towards floral tissue, respectively. These findings advance our understanding of the hormonal regulation of sex expression in *C. sativa* and identify optimized approaches for its manipulation.

## 1. Introduction

*Cannabis sativa* is a dioecious annual angiosperm (Cannabaceae) domesticated for fiber, seed, and cannabinoid production [1,2,3]. Historical restrictions on cultivation have limited the application of modern molecular breeding approaches compared to other crop species, and the relatively recent relaxation of these restrictions have now created significant opportunities for crop improvement [4,5]. Sex expression is a critical economic trait in *C. sativa*, as its product value depends on sex ratios: males produce superior fiber, whereas females generate high cannabinoid yields and a higher proportion is favored for seed production [6,7]. Deviations from optimal sex ratios reduce yield and complicate harvest timing [8]. Consequently, gaining a deeper understanding of, and control over, sex determination is essential for improving crop performance [5].

*C. sativa* exhibits a highly plastic morphology influenced by genetic and environmental factors, including flowering phenotypes [9]. Age-dependent axial flowers (ADFs) form at 3–5 nodes, whereas a reduced photoperiod triggers a transition to reproductive development, producing large photoperiod-dependent inflorescences (PDIs) [10,11]. ADFs and PDIs have also been referred to as “botanical pistillate flowering” and “commercial pistillate flowering”, respectively [12]. Males are typically taller, flower earlier, and form branched cymose panicles that release pollen at anthesis [13,14]. Females develop compound racemes with bract-enclosed flowers bearing exserted stigmas [15]. Sexes differ physiologically and anatomically, notably in glandular trichome density, with females producing abundant cannabinoid- and terpenoid-rich trichomes derived from MEP/MVA pathways [16,17], while vascular differences contribute to superior male fiber quality [8,18].

Unisexual flowers, as seen in *C. sativa*, are rare among angiosperms, occurring in approximately 10% of species [19]. *C. sativa* exhibits diverse sexual phenotypes, including dioecy, bred monoecy, and occasional floral hermaphroditism, reflecting complex sex determination and expression mechanisms [20,21,22,23]. The species is diploid (2n = 20), with heteromorphic sex chromosomes: males are XY and females are XX [24,25]. The Y chromosome is larger, resulting in a greater genome size in males [26]. Despite chromosomal determination, *C. sativa* exhibits pronounced sexual phenotypic plasticity and readily undergoes sex reversal (the development of flowers of the opposing sex to sex chromosome make-up) in response to environmental, pathological, or experimental factors [27].

Hormones are widely recognized as central regulators of sex differentiation and expression in plants, providing a mechanistic interface between environmental cues and underlying genetic controls [27], and most major plant hormone classes have been shown to influence sex differentiation or fertility to varying degrees [28]. Plant growth regulators (PGRs), which modulate endogenous hormone signaling, have therefore been extensively used to investigate and manipulate sex expression, offering both practical control and insight into regulatory mechanisms [27]. In *C. sativa*, numerous studies have demonstrated that chemical treatments can induce sex reversal using a range of compounds and application methods (summarized in Supplementary Table S1). The majority of these studies have focused on PGRs targeting gibberellin (GA) and ethylene pathways, two hormone classes long implicated in plant sex determination [29]. In *C. sativa*, these hormones reportedly act antagonistically: GA promotes male flower development in female plants, whereas ethylene promotes female flower development in males [30]. More recent studies have begun to identify the central role ethylene has in *C. sativa* sex reversion and sex determination more broadly [31,32,33,34].

Other hormone classes, including cytokinins, auxins, and abscisic acid (ABA), have also been examined. Cytokinins and auxins have been reported to exert feminizing effects on male *C. sativa* plants [35], while ABA appears to primarily suppress the effects of other exogenously applied PGRs rather than inducing sex reversal independently [30]. These responses are not conserved across species; for example, the cytokinin zeatin induces masculinization in *Mercurialis annua* [36]. The induction of male flowers on genetically female *C. sativa* plants, followed by crossing or selfing, yields 100% female progeny and underpins the commercial production of feminized seed [37,38]. In contrast, the fertility of ethylene-induced female flowers on male plants has only recently been reported [39].

Whether auxins, cytokinins, ethylene, and gibberellins directly regulate sex expression in *C. sativa* or act indirectly through hormonal cross-talk with ethylene remains poorly understood, although the inhibition of individual pathways is often assumed to elicit opposing effects. Experimental outcomes are further confounded by variations in PGR delivery methods and developmental timing. Foliar application after visual sex determination is most common, producing strong, localized effects lasting approximately 4–6 weeks [40]. In contrast, systemic approaches, including seed soaking, root immersion, and apical wicking, have generated hermaphroditic flowers and intersex phenotypes [30,41,42]. Early seedling treatments reportedly lacked the transient response observed in mature plants, suggesting strong developmental effects [10]. Notably, gibberellic acid (GA3) seed pretreatment reportedly increased female frequency, opposing its masculinizing effects in mature plants [42].

Although these studies have clearly demonstrated that sex reversal in *C. sativa* can be induced throughout development using various plant growth regulators (PGRs), the interactions among hormone classes regulating sex expression remain poorly understood. In particular, it is unclear whether a single primary hormone pathway governs sex determination or whether individual hormone classes act through distinct mechanisms. Collectively, methodological heterogeneity, genetic variability, and limited direct comparisons have obscured identification of the most influential hormonal pathways and optimal treatment strategies. The effects of PGR application on fertility also require clarification. While silver thiosulfate (STS)-induced pollen and ethephon (ETH)-induced ovaries are known to be viable, the hormonal regulation of ovule fertility and pollen viability have not been investigated, despite their potential value for breeding programs [37,43]. A systematic comparative evaluation of sex reversion techniques in *C. sativa* is therefore required.

This study systematically examines the hormonal control of sex expression by applying nine different compounds that stimulate or inhibit major hormone pathways, individually and in combination, via foliar sprays (Table 1). Treatments were repeated on four genotypes (*n* = 6), male and female clones from a high-CBD drug-type accession from Syria (Syr_M, Syr_F), and a hempseed accession from Romania (Rom_M, Rom_F). By controlling for genotype and treatment methodology, this approach enables a direct comparison of hormonal effects and advances the understanding of scalable sex manipulation strategies.

## 2. Results

### 2.1. Establishment of Treatment Regimes

As the majority of the PGRs used in this study had either not been used on *C. sativa* or not used as a foliar spray, initial small-scale dosage trials were undertaken on either the Rom_F or Rom_M clone to determine the most appropriate treatment methodology (Supplementary Table S2). A matrix of three concentrations and up to three applications were tested if PGRs had not been used as a foliar spray in Cannabis, while PGRs with clear literature precedence (i.e., STS and ETH) required reduced treatment optimization. In these initial dosage trials, where sex reversion did not occur, the strongest dosage that did not result in major detrimental effects was chosen for the subsequent comparisons across genotypes (Table 1).

### 2.2. Effect on Flower Type and Genotype Specificity

**Figure 1:**
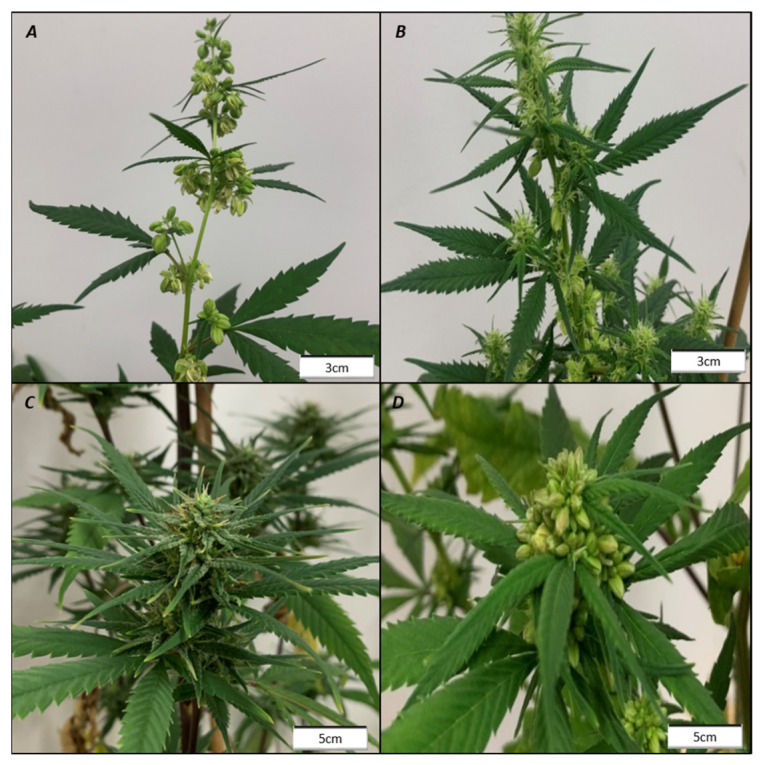
Inflorescence structure is unaffected by sex reversion. An untreated Syr_M clone (**A**) and an ethephon (ETH)-treated Syr_M clone showing male and female flowers (**B**) both exhibiting panicle inflorescences. An untreated Rom_F clone (**C**) and a silver thiosulfate (STS)-treated Rom_F clone with male flowers (**D**) both exhibiting a compound raceme inflorescence. Photos taken 21 days after reducing the photoperiod to 12:12.

**Figure 2:**
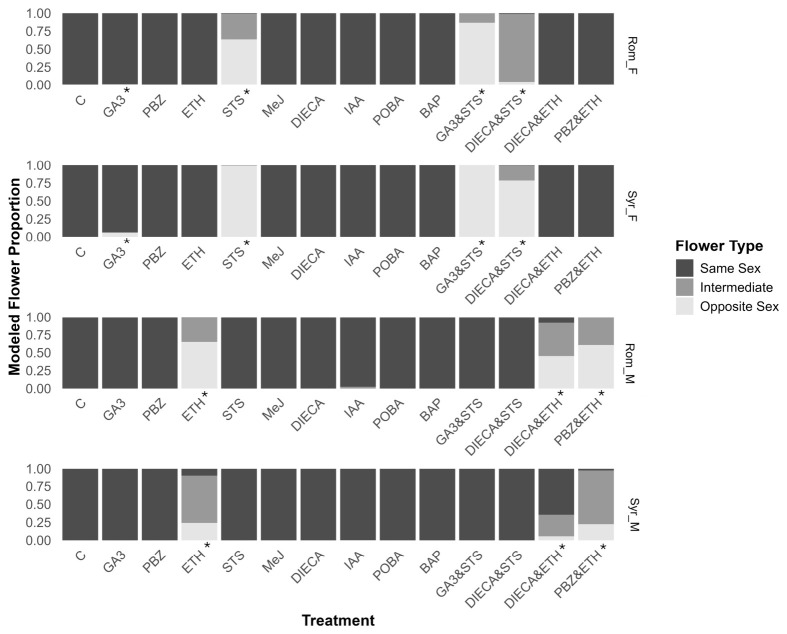
Effectiveness of treatments’ capacity to induce sex reversal. Modeled proportions of same sex, opposite sex and intermediate inflorescences of total floral biomass for experimental treatments. * Signifies meaningful difference from control. Compounds used in treatments include de-ionized water (control), gibberellic acid (GA3), paclobutrazol (PBZ), ethephon (ETH), silver thiosulfate (STS), methyl jasmonate (MeJ), diethyl dithiocarbamate (DIECA), indole acetic acid (IAA), phenoxyphenyl boronic acid (POBA), and 6-benzylamino purine (BAP). Refer to Supplementary Table S3 for individual posterior distributions and 95% HPDIs (highest posterior density intervals).

The hormone treatments had comparable effects in both genotypes tested but markedly different effects depending on the sex. In treatments where sex reversion did occur, the inflorescence structure was not altered, with female plants exhibiting compound racemes and males cymose panicles as expected for each sex (Figure 1). The single treatments of paclobutrazol (PBZ), methyl jasmonate (MeJ), diethyl dithiocarbamate (DIECA), phenoxyphenyl boronic acid (POBA), and 6-benzylamino purine (BAP) had no direct effects on sex expression and did not instigate the reversion of sexual phenotypes in any of the tested genotypes (Figure 2), whereas gibberellic acid (GA3), ethephon (ETH), silver thiosulfate (STS), indole acetic acid (IAA), and their combination induced sex reversion in either males or females of the genotypes trialed (Figure 2).

The trends among the treatments that induced opposite flowers and hermaphroditic inflorescences were mostly consistent in both accessions. However, the Syrian_F accession was more amenable to the female-to-male sex reversion, while the Rom_M was the most amenable to male-to-female sex reversion (Figure 2).

### 2.3. Reversion of XX Female to Male Phenotype

**Figure 3:**
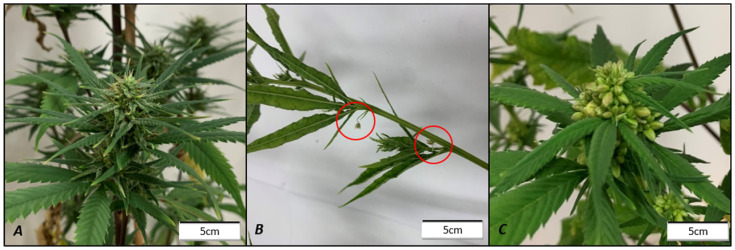
Difference in location of male flowers in GA3 and STS treatments on Syr_F. (**A**) Control, showing no male flowers. (**B**) Gibberellic acid (GA3); male flowers circled in red at axial nodes typical of ADF, while racemose inflorescence remained female. (**C**) Silver thiosulfate (STS); male flowers throughout racemose inflorescence typical of PDI, while ADFs at axial nodes remained female. Photos taken 21 days after reducing the photoperiod to 12:12.

The growth of male flowers on female plants was instigated in both female genotypes through GA3 and STS treatments (Figure 2). STS treatment had a larger effect than GA3, resulting in a greater probability of producing male flowers. STS produced a maximum effect in Syr_F, with a probability of 0.99 (95% CI 0.991–0.993) of producing male flowers, while the GA3 treatment had a probability of only 0.001 (95% 0.0006–0.0013) in Rom_F (Supplementary Table S3). It was observed that STS treatments caused the reversion of PDIs to male flowers while ADFs remained female, whereas GA3 treatments only instigated sex reversion of ADFs at axial positions, while PDIs remained female (Figure 3). Thus, the combination of these compounds had a complementary effect, resulting in the treatment using these compounds in combination having the greatest effect on sex reversion, with median probabilities of 0.86 (95% CI 0.854–0.877) and 0.99 (95% CI 0.997–0.998) for Rom_F and Syr_F accessions, respectively.

While the DIECA treatment did not affect flower sex in females by itself, it reduced the probability of male flowers and increased the probability of hermaphrodite inflorescences when applied with STS (Figure 2). This was most apparent in Rom_F, with the median probability of opposite flowers being produced dropping from 0.63 (95% CI 0.625–0.645) to 0.04 (95% CI 0.042–0.048), and hermaphroditic inflorescences increasing from 0.35 (95% CI 0.346–0.366) to 0.94 (95% CI 0.937–0.944).

### 2.4. Reversion of XY Male to Female Phenotype

In both male genotypes, only treatments of ETH or ETH in combination (treatments DIECA and ETH, and PBZ and ETH) were able to instigate the development of female flowers (Figure 2). ETH treatment of Rom_M resulted in the greatest probability of producing female flowers, with a median probability of 0.65 (95% CI 0.634–0.668). IAA did also influence sex expression, however, with a small probability that hermaphroditic flowers were produced after treating male genotypes (Figure 2). All observed female and intermediate flowers were of the PDF type, whilst all ADFs appeared resistant to PGR treatments and remained male.

The combination of ETH and PBZ on male plants increased the probability of producing hermaphroditic flowers at the expense of both male and female flowers compared to the single ETH treatment (Figure 2). When DIECA and ETH were applied together, however, there was a substantial increase in the likelihood of male flowers at the expense of female and hermaphroditic flowers for Syr_M compared to ETH alone. The probability of hermaphroditic inflorescences decreased from 0.66 (95% CI 0.648–0.682) to 0.30 (95% CI 0.287–0.308) in Syr_M, while it slightly increased from 0.34 (95% CI 0.328–0.362) to 0.47 (95% CI 0.447–0.485) in Rom_M.

### 2.5. Fertility of Reverted Flowers

**Figure 4:**
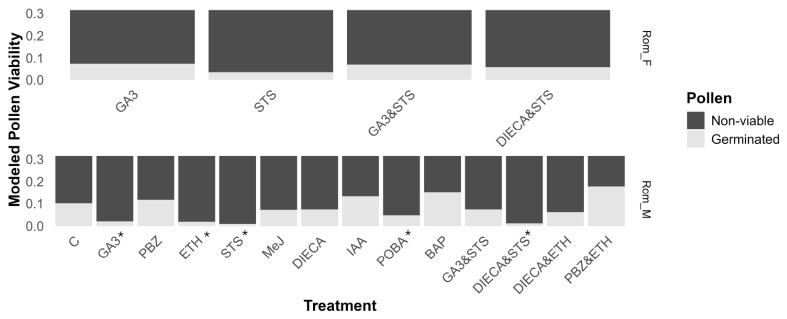
Effect of treatments on pollen viability. Modeled proportion of viable pollen from the Rom accession. Pollen was considered viable if a pollen tube was observed after 18 h of incubation in pollen media. * Signifies meaningful difference from control. Compounds used in treatments include de-ionized water (control), gibberellic acid (GA3), paclobutrazol (PBZ), ethephon (ETH), silver thiosulfate (STS), methyl jasmonate (MeJ), diethyl dithiocarbamate (DIECA), indole acetic acid (IAA), phenoxyphenyl boronic acid (POBA), and 6-benzylamino purine (BAP). Refer to Supplementary Table S3 for individual posterior distributions and 95% HPDIs (highest posterior density intervals).

Syrian pollen was not assessed due to its low germination rates in the control (1.2%) and previous breeding experiments showing that it produces poor-quality pollen (unpublished data). No difference in pollen viability was observed in the treatments that caused the development of male flowers in Rom_F (Figure 4). On the other hand, there was a great variation in pollen viability in Rom_M, with the PBZ&ETH treatment having the highest viability, at 17.8% (95% CI 14.3–21.8), although the majority of treatments reduced pollen viability compared to the control (Figure 4). Interestingly, the single or combined application of masculinizing compounds (GA3 and STS) caused strong decreases in pollen viability in Rom_M, as did the single ETH treatment, while adding PBZ to the ETH treatment restored the pollen fertility (Figure 4). Fertilized female flowers were observed in all ETH treatments of Rom_M and Syr_M.

### 2.6. Pleiotropic Effects

All 13 treatments produced distinct phenotypes, with some morphological changes not associated with sex expression. These pleiotropic effects are summarized in Table 2, and combination treatments exhibited a mix of these phenotypes. However, the most distinct of these changes were on plant height, with GA3 treatments increasing height in all genotypes, whereas PBZ, ETH and MeJ had dwarfing effects, albeit the effect of MeJ was very short-lived, and the heights of this treatment group recovered quickly (Supplementary Table S4). Additionally, some treatments produced fairly phytotoxic effects, with DIECA causing peripheral leaf burn, IAA causing swollen stems and BAP causing dieback of shoots.

### 2.7. Flower Developmental Timelines and Biomass

**Figure 5:**
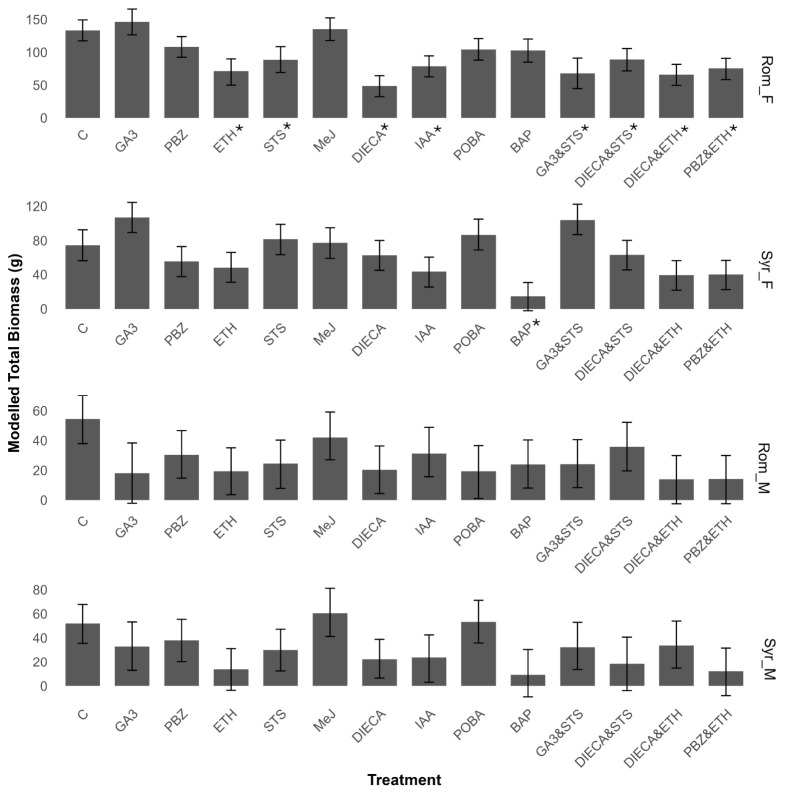
Effect of treatments on total biomass (g). Modelled total aboveground biomass in grams. Error bars are shown as 95% CI range. * Signifies meaningful difference from control. Compounds used in treatments include de-ionized water (control), gibberellic acid (GA3), paclobutrazol (PBZ), ethephon (ETH), silver thiosulfate (STS), methyl jasmonate (MeJ), diethyl dithiocarbamate (DIECA), indole acetic acid (IAA), phenoxyphenyl boronic acid (POBA), and 6-benzylamino purine (BAP). Refer to Supplementary Table S3 for individual posterior distributions and 95% HPDIs (highest posterior density intervals).

In both accessions, the female’s floral development was slower, reaching anthesis 5 days later than the males, resulting in a later harvest. Rom_F and Syr_F both had larger biomasses compared to their male counterparts (Figure 5). No treatment meaningfully increased total aboveground biomass, and, in general, the biomass of male plants was more consistent among treatments, with no treatment meaningfully altering total biomass in either Rom_M or Syr_M (Figure 5). The total biomass of Rom_F was the most labile, with all treatments reducing total biomass except for GA3, PBZ and MeJ (Figure 5), whereas BAP reduced total biomass compared to the control in Syr_F from 74.5g (95% CI 56.61–92.56) to 14.7g (95% CI 0.0–30.61).

### 2.8. Reproductive Resource Allocation

**Figure 6:**
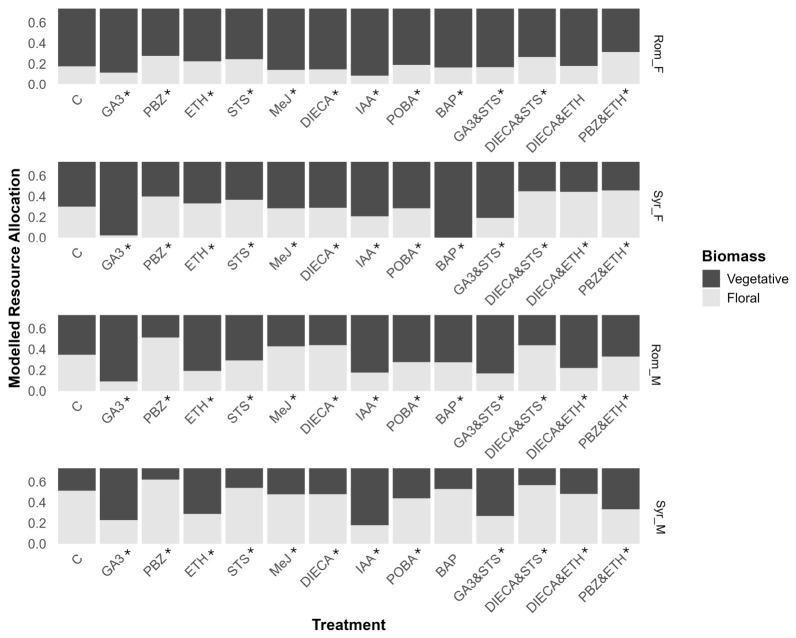
Effect of treatments on reproductive resource allocation (RRA). Modelled RRA calculated as the proportion of total biomass in male or female flowers. Median with posterior distributions in black. * Signifies meaningful difference from control. Compounds used in treatments include de-ionized water (control), gibberellic acid (GA3), paclobutrazol (PBZ), ethephon (ETH), silver thiosulfate (STS), methyl jasmonate (MeJ), diethyl dithiocarbamate (DIECA), indole acetic acid (IAA), phenoxyphenyl boronic acid (POBA), and 6-benzylamino purine (BAP). Refer to Supplementary Table S3 for individual posterior distributions and 95% HPDIs (highest posterior density intervals).

Reproductive resource allocation (RRA) was calculated by dividing the total floral biomass by the total aboveground biomass as a measure of harvest index and total reproductive capacity. Despite having lower total biomasses, male plants of both accessions had a higher RRA than females, except where treatments induced sex reversion in the males (treatments ETH, DIECA and ETH, and PBZ and ETH) and in the GA3&STS combination treatment (Figure 6). Additionally, where sex reversion occurred, the transition from the female to male phenotype via STS application increased RRA, whereas the change from male to female flowers via ETH reduced overall RRA.

The gibberellin pathway strongly influenced resource allocation in all genotypes, with its promotion via GA3 treatment greatly reducing RRA and its inhibition by PBZ increasing RRA when compared to the control (Figure 6). GA3’s effect on RRA was most pronounced in Syr_F, causing the RRA to drop from 0.3 (95% CI 0.296–0.305) for the control to 0.23 (95% CI 0.223–0.248) when treated with GA3, whereas the largest increase in RRA was for the PBZ&ETH dual application in Rom_F, increasing RRA from 0.17 (95% CI 0.173–0.179) for the control to 0.31 (95% CI 0.311–0.319).

## 3. Discussion

### 3.1. Pathways for Female-to-Male Sex Reversion

The instigation of female-to-male sex reversion, resulting in fertile pollen, was possible via the manipulation of two hormone pathways: the promotion of the gibberellin pathway via GA3 application, and the inhibition of the ethylene pathway via STS (Figure 2). Female-to-male sex reversion is more studied in *C. sativa* due to its use in creating feminized seed, and STS is currently the most common PGR treatment used to do this [38]. The results in this study align with previously reported effects of these compounds in *C. sativa* [31,37,43,44]. GA3 and STS have also been shown to instigate the development of male flowers in gynoecious *Cucumis sativus* [45], suggesting similar convergent or conserved mechanisms.

Because GA3 reversed the ADFs and STS reversed the PDI flowers, the combination of STS and GA3 resulted in the most complete female-to-male sex reversion of 86 to 100%. This is, to our knowledge, the first example in *C. sativa* of multiple PGRs instigating a more complete sex reversal. However, GA3 treatment also resulted in a lower RRA (Figure 6). Therefore, using only STS would be a more appropriate treatment to instigate male-to-female sex reversion in Syr_F, where conversion was already near-complete for this treatment, whereas the combination may be advantageous for treating Rom_F, where STS alone resulted in a large proportion of hermaphroditic flowers, highlighting the variability of genotypes’ responses to PGR treatment. Moreover, the ability to induce complete sex reversion can be highly valuable from a research/breeding perspective.

### 3.2. Pathways for Male-to-Female Sex Reversion

Of all the treatments tested, only the promotion of the ethylene pathway via the application of ETH was able to induce male-to-female sex reversion in PDIs (Figure 2). This confirms previous work in *C. sativa*, where the stimulation of the ethylene pathway resulted in the feminization of males [40,46,47,48]. A similar effect has been reported in *Curcurbita moschate*, where the application of ETH stimulated female flower formation [49].

Interestingly, despite gibberellin pathway promotion in females resulting in male ADF, its inhibition via PBZ application did not produce female ADF on male plants (Figure 2). However, the dual application of PBZ and ETH reduced the likelihood of producing male flowers on male genotypes to a greater degree than just ETH on its own, indicating that PBZ did have a feminizing effect on PDIs when applied in combination with ETH.

Female flowers are *C. sativa*’s highest-value product, being the site of therapeutically important cannabinoid biosynthesis [16]. As such, a greater understanding of the underlying molecular networks of female flower formation has direct economic implications. While the female-to-male sex reversion in an XX background has been investigated more thoroughly [31,33], the underlying molecular changes in ETH treatments have only recently been investigated [32,34] and represent a unique opportunity for understanding female flower formation. The observed seed setting in this study indicates that fertility is maintained in the ETH-induced female flowers despite the plants being XY. The potential for XY plants to set seed via ETH treatment was explored in a previous study [39], where the shorter developmental timeline was found to be advantageous in an inbreeding context, and opens the possibility of better targeting male-specific traits in breeding programs.

### 3.3. Hormonal Control of Sex Reversion

The results of this study suggest that sex expression in *C. sativa* is primarily controlled by ethylene signaling. This was shown by the effective masculinization of female clones through the inhibition of ethylene biosynthesis via STS treatment and the feminization of male clones through the promotion of ethylene levels via ETH treatment (Figure 2). Previous studies showed that many ethylene-related genes are on the X-chromosome [33], creating the potential for dosage effects in the ethylene biosynthesis pathway with downstream effects on sex expression. However, sex-reverted flowers in both treatments only occurred in PDIs. On the other hand, the promotion of the gibberellin pathway via GA3 treatment resulted in the reversal of female ADFs only and had a large impact on the overall production of sex organs through alterations in RRA, resulting in a very different phenotype to STS treatment (Figure 3). Previous studies have similarly described the formation of male flowers in axial nodes as a result of GA3 treatment [30,50]. The finding that GA3 induced male flowers in female clones, whereas the reverse did not occur with PBZ, may indicate that the masculinization observed with GA3 is at least partially due to its inhibition of ethylene biosynthesis, as Dugardeyn et al. (2008) reported that gibberellins can impact the availability of ethylene precursor 1-amino-cyclopopane-1-carboxylic acid (ACC) [51]. While reducing gibberellin availability, PBZ application in males did not increase ethylene levels and, therefore, did not instigate sex reversion (Figure 2). The ethylene and gibberellin pathways are known to influence each other in a range of different processes such as transition to flowering and seed dormancy, as ethylene has been shown to stabilize DELLA proteins, the negative regulators of gibberellin signaling, against gibberellin-mediated degradation [52]. However, the finding that STS alone was not able to reverse the sex of ADFs, and that ABA is known to antagonize GA3 masculinization [30], suggests that their reversal is not solely due to the GA3 effect on ethylene signaling. This indicates two distinct regulatory pathways for ADF and PDI that could potentially mirror cucurbits, where sex can be manipulated by altering ethylene levels using PGRs and GA3 [49], and the expression of ethylene biosynthesis genes determines sex [53]. The results of this study draw many parallels between the control of sex expression in *C. sativa* and cucurbits, providing a useful basis for further study into *C. sativa* sex expression.

### 3.4. Hormone Pathways Not Useful for Altering Sex Expression

In their review, Chandler et al. [28] provide evidence that most major hormone classes affect sex expression and fertility in plants to some degree, and previous publications in *C. sativa* indicate that the modulation of auxins and cytokinins affects sex expression [30,41,44]. However, this study demonstrates that the role of auxins and cytokinins in *C. sativa* sex expression is minimal. While tissue-specific auxin biosynthesis has been shown to be an important regulator of sex organ development in Arabidopsis, Maize and Rice [28], the inhibition of IAA’s biosynthesis through POBA treatment in floral and meristematic tissue had no measurable effect on sex expression. Apart from instigating the formation of a very small number of intermediate inflorescences in the male genotypes (Figure 2), this study found the foliar application of IAA ineffective for sex reversion, contrasting with previous reports using root immersion of seedlings or wicking of the apical bud of seed grown material post-flower induction [10,30,35].

Since IAA and ethylene are known to positively interact [54], the intermediate inflorescences observed in IAA-treated plants may be due to an increase in endogenous ethylene levels. The ethylene pathway is strongly involved in the development and senescence of plants, as well as sex expression in *C. sativa*, with Spitzer-Rimon et al. (2022) reporting changes in the regulation of several ethylene-responsive factors during a female *C. sativa*’s transition to sexual maturity [55]. Thus, the effect of age and developmental stage may explain the differences between this study and the previously reported effects of these compounds, and the response of *C. sativa* to PGR treatments across different stages of development merits further research. Furthermore, the only observable impact of cytokinin promotion via BAP application was the inhibition of flowering in Rom_M (Figure 2), despite previously being reported to have a feminizing effect on male *C. sativa* [30].

Additionally, Adal et al. (2021) did find genes associated with multiple hormone pathways differentially expressed after STS-induced sex reversal, with auxin, cytokinin and gibberellin pathways being affected [31]. However, the results of this study would suggest that these pathways have a minor effect on sex expression, and the alterations seen in Adal et al. (2021) may reflect the STS treatment’s pleiotropic effects [31].

Although DIECA did not induce sex reversion in any of the genotypes, its combination with either STS or ETH reduced the prevalence of opposite sex flowers and increased intermediate flowers (Figure 2). This seemingly non-specific inhibition of sex reversion is akin to the reported effect of ABA [30]. However, whether this effect is specifically due to known interactions between jasmonate and ethylene signaling [56] or due to the jasmonate pathway directly influencing sex expression requires further investigation.

### 3.5. Pleiotropic Effects and Resource Allocation

Due to the wide-ranging effects of hormones on plant growth and development, most of the compounds used in this study elicited phenotypic responses in the treated clones outside of sex reversion (Table 2). Many reflected classical impacts of these pathways, such as auxin’s role in apical dominance represented by increased branching in POBA-treated plants [57] and the shortened internode length of ETH-treated plants [58], but also included phytotoxic and necrotic effects. As a result, many of the treatments within this study altered the resource allocation of the plants, while no treatments meaningfully increased the total biomass compared to the control (Figure 5).

The male phenotypes had overall higher RRA than the females (Figure 6), which is consistent with the higher energetic cost of producing ovules and seeds compared to pollen, as males only need to exert energy while pollen are developing, while females require a consistent energetic outlay to maintain the growing seed [59].

The main induced effect on resource allocation was linked to a manipulation of the gibberellin pathway, showing an increase in RRA in response to PBZ and a decrease in response to GA3 treatment. This was expected, as past increases in the harvest index, a major driver of yield increases since the Green Revolution [60], have traditionally been achieved through a manipulation of the gibberellin pathway. Breeding to increase the harvest index has primarily been achieved by downregulation through the inactivation of gibberellin biosynthesis enzyme gibberellin oxidases, whereas PBZ achieves downregulation through the inhibition of kaurene oxidase, an enzyme from earlier in the biosynthetic pathway [61]. The inhibition of this oxidase via genetic means is likely problematic due to the importance of gibberellins in other biological processes such as seed germination [62], but the application of PBZ during the onset of flowering has been postulated to increase RRA, which could increase seed and cannabinoid yield in *C. sativa*, while GA3 application may have application in fiber production contexts [63]. Our results support these postulations. However, the strong negative effect of GA3 on the RRA reduces the scope of this compound for applications such as feminized seed production.

### 3.6. Genotype Effects

The treatments tested in this study had markedly different effects depending on the plant’s sex and broadly similar effects when comparing the same sex of the two different accessions. This is despite the accessions originating from diverse locations and having different end uses. While the majority of previous research into *C. sativa* sex reversion has used a single accession/population, this observation mirrors the differing degrees of STS-induced masculinization reported by Lubell et al. (2018) and reinforces the importance of genotype-specific PGR treatments when inducing sex reversion in *C. sativa* [37].

In terms of each accession’s capacity for sex reversion after PGR treatment, the Romanian accession showed a greater propensity to stay/shift towards the female phenotype, with a greater proportion of female flowers post-ETH treatment in Rom_M and a lower proportion of male flowers post-STS treatment in Rom_F (Figure 2), whereas the Syrian accession was the opposite (Figure 2). This indicates that there may be some underlying differences in hormone transmission (particularly ethylene) between the accessions. Identifying whether this is due to differences in the expression of biosynthesis genes or increased sensitivity in signal perception and transduction would require further gene expression studies. For example, the co-expression of copper transporters has been reported to increase ethylene sensitivity and signal transduction by lowering ethylene receptor binding restraints [64]. How potential differences in underlying hormone transmission relate to monoecious sexual phenotypes in *C. sativa* requires further investigation [65].

### 3.7. Conclusions

This study comprehensively compared the capacity of different PGRs to induce sex reversion in *C. sativa* via foliar applications on male and female clones of two distinct accessions. To our knowledge, this is the first study to consistently explore sex-specific PGR effects on both sexes in multiple accessions. We used this methodology to optimally harness the ability to induce sex reversion in *C. sativa* and to gain improved insight into each hormone pathway’s effect on sex reversion on both male and female clones. The results show that the most effective method to induce sex reversal is through the manipulation of the ethylene pathway that allows for the formation of fully fertile flowers of the opposite sex. Furthermore, we showed that the age-dependent axial flowers (ADFs) of female plants can only be reversed via the gibberellic pathway, and that complete sexual reversal therefore requires manipulation of both the ethylene and gibberellic pathways. The finding that the sex determination of ADFs and photoperiod-dependent inflorescences (PDIs) is controlled differently opens further possible inquiries into the underlying regulatory networks of flower formation and sex expression.

## 4. Materials and Methods

### 4.1. Plant Materials

Cannabis cultivation, sampling, storage and processing were performed in strict adherence to the obligations of the low-THC hemp license (no. 52204) issued to Southern Cross University by the New South Wales Department of Primary Industries and Regional Development (Australia). Cannabis germplasm (IPK_CAN_57) was obtained from the Leibniz Institute of Plant Genetics and Crop Plant Research (Leibniz-Institut für Pflanzengenetik und Kulturpflanzenforschung (IPK), Seeland, Germany), imported under a federal Office of Drug Control (ODC) license to import No. 1820928, and handled under the Food and Agriculture Organization of the United Nations governed Standard Material Transfer Agreement (sMTA).

Seeds from IPK_CAN_57 (a CBD-dominant drug-type accession from Syria named “Syr”) and IPK_CAN_32 (a seed accession from Romania named “Rom”) were germinated on paper towels at 26 °C for 5 days before germinated seeds were planted into hydroponic media (70% coco coir, 30% perlite) supplemented with Osmocote Pro-3-4M (4.3 g/L) for a further ten days. Sex was determined by visual inspection of ADFs before stock plants were then selected from a single male and female from both lines, representing four genotypes referred to as Rom_M, Rom_F, Syr_M, and Syr_F.

Stock plants were grown at 26 °C under LED lights (300–450 µmol/m^2^/s) with an 18:6 h photoperiod in hydroponic media (70% coco coir, 30% perlite, Garden Mediums Pty. Ltd., Ballina, Australia) supplemented with Osmocote Pro-3-4M (4.3 g/L). Cuttings were taken using Eazy Plug propagation trays and Clonex (3000 ppm), with an 18:6 h photoperiod (100–150 µmol/m^2^/s), before being potted up after 10 days. Rooted cuttings were grown vegetatively for four days at 26 °C under the same conditions as the stock plants before experimental treatments began. Syr_F/M took longer to root and establish. Thus, an extra 10 days were given in the vegetative stage.

### 4.2. Plant Growth Regulator Treatments

Nine plant growth regulators (PGRs) from five different hormone families, a control and four two-way PGR combinations were selected for this trial (Table 1). PGRs were sourced from Austratec Pty Ltd. (Melbourne, Australia)—gibberellic acid (GA3), paclobutrazol (PBZ), methyl jasmonate (MeJ), and phenoxyphenyl boronic acid (POBA)—or Sigma Aldrich Australia Pty Ltd. (Sydney, Australia)—diethyl dithiocarbamate (DIECA), ethephon (ETH), silver thiosulfate (STS), indole acetic acid (IAA), and 6-benzylaminopurine (BAP). Concentrated stock solutions were made by dissolving PGRs in water (ETH, STS), ethanol (PBZ, MeJ, IAA), 0.1M NaOH (GA3, DIECA, BAP) or DMSO (POBA) before working solutions were made at the required concentrations with de-ionized water (Table 2). Working solutions were supplemented with 0.1% Tween20. The control treatment contained de-ionized water and 0.1% Tween20.

As most of the PGRs had not previously been applied to *C. sativa* or had not been evaluated as foliar applications, preliminary small-scale dose–response trials were conducted to establish appropriate treatment conditions (Supplementary Table S2). In these preliminary trials, up to three different concentrations in up to five applications were used on a single clone to determine the most effective treatment strategy. Rom_F was used for GA3 and POBA optimization, while Rom_M was used for PBZ, MeJ, DIECA, ETH, IAA and BAP optimization. Where preliminary trials did not induce sex reversion, the highest dose that did not produce substantial adverse effects was selected for subsequent experiments.

To compare treatments across the genotypes, six clones were allocated to each of the fourteen treatments per genotype (*n* = 6); however, some losses were recorded (Supplementary Table S5). The first treatment (Day 1) was applied 6 days before the light regime was changed to induce flowering. Foliar treatments were applied at the end of the light cycle, with exhaust fans switched off to allow maximum contact with the leaves. Upon application, each plant was thoroughly coated until dripping (~10 mL). Flowering was induced on Day 7 by introducing plants to a 12:12 h photoperiod under HPS lights (1250 µmol/m^2^/s), and each treatment was isolated in individual growth chambers during flowering.

### 4.3. Harvest Measurements

Harvest occurred when 50% of the male flowers reached anthesis. Aboveground biomasses were separated into male, female, and intermediate inflorescences, as well as non-reproductive biomass (stems and leaves). Inflorescences were deemed intermediate if they contained either hermaphroditic flowers and/or a mix of male and female flowers.

Pollen samples were harvested from Rom_M and Rom_F treatments where male flowers were produced. Three micro-centrifuge tubes (*n* = 3) containing pollen in liquid germination media (17% sucrose, 30 mgL^−1^ Ca(NO_3_)_2_, 100 mgL^−1^ H_3_BO_3_, pH 6.4) as per the methodology of DiMatteo and colleagues (2020) were incubated without the gelling agent for 18 h at 26 °C per treatment. After this, 200 pollen grains per tube were scored for germination, as signified by the appearance of a pollen tube.

### 4.4. Statistical Analysis

A Bayesian multinomial regression model was fitted in the brms package (v2.22.0) [66] in R v4.2.2 [67]. A Bayesian analytical approach was used due to frequentist tests’ sensitivities to small sample size and the potential for type II errors induced by post hoc comparisons due to the large number of experimental treatments used in this study [68,69].

#### 4.4.1. Flower Proportions

For each plant, the probabilities of a gram of floral biomass being (1) of the opposite sex and (2) hermaphroditic were modelled, and each treatment and plant sex (male/female) combination with a further interaction effect of accession (Rom/Syrian) and sex was modelled separately. In brms syntax, the model formula was thus:

y | trials(N) ~ 0 + Treatment × Sex + Accession × Sex

where N is the total floral biomass of each plant. The accession and sex interaction was included after seeing the plotted data.

#### 4.4.2. Pollen Viability

For each plant and each treatment and plant sex (male/female) combination, the probabilities of a pollen grain being (1) viable/germinated were modelled. In brms syntax, the model formula was thus:

y | trials(N) ~ 0 + Treatment × Sex

where N is the probability of a pollen grain being viable.

#### 4.4.3. Reproductive Resource Allocation

For each plant, the probabilities of a gram of biomass being (1) floral and (2) vegetative were modelled, and each treatment and plant sex (male/female) combination with a further interaction effect of accession (Rom/Syrian) and sex was modelled separately. In brms syntax, the model formula was thus:

y | trials(N) ~ 0 + Treatment × Sex + Accession × Sex

where N is the total biomass of each plant. The accession and sex interaction was included after seeing the plotted data.

Student-*t*_4_(0, 3) priors were used for coefficients because this places most of the prior mass near 0 and 1 on the probability scale, where most probabilities were expected to occur, and allows for some extreme values. Four chains of 2000 iterations were run after warm-up periods of 2000 iterations. Predicted posterior distributions for each “treatment × sex × accession” combination were summarized with medians and 95% highest posterior density intervals (HPDIs), and were considered meaningful when they did not overlap. Plots were generated using ggplot2 (v3.5.0) [70,71].

## Figures and Tables

**Table 1:** Summary of experimental treatments. First treatment began 6 days before changing the photoperiod from 18:6 to 12:12 to induce flowering. Compounds used in treatments include de-ionized water (control), gibberellic acid (GA3), paclobutrazol (PBZ), ethephon (ETH), silver thiosulfate (STS), methyl jasmonate (MeJ), diethyl dithiocarbamate (DIECA), indole acetic acid (IAA), phenoxyphenyl boronic acid (POBA), and 6-benzylamino purine (BAP). Refer to Supplementary Table S2 for regimes tested in initial dosage trials. ↑ and ↓ refers to promotion and inhibition of the pathway, respectively.

Plant Growth Regulator	Major Effect on Hormone Pathway	Concentration [mM]	Number of Applications
De-ionized water (control)	NA	NA	5
Gibberellic acid (GA3)	↑ Gibberellins	0.58	5
Paclobutrazol (PBZ)	↓ Gibberellins	0.14	3
Ethephon (ETH)	↑ Ethylene	4.3	2
Silver thiosulfate (STS)	↓ Ethylene	6	3
Methyl jasmonate (MeJ)	↑ Jasmonates	0.2	5
Diethyl dithiocarbamate (DIECA)	↓ Jasmonates	50	4
Indole acetic acid (IAA)	↑ Auxins	1.1	3
Phenoxyphenyl boronic acid (POBA)	↓ Auxins	0.02	5
6-benzylamino purine (BAP)	↑ Cytokinins	0.2	3
DIECA & STS	↓ Jasmonates + ↓ Ethylene	50 and 6	4 and 3
DIECA & ETH	↓ Jasmonates + ↑ Ethylene	100 and 4.3	4 and 2
PBZ & ETH	↓ Gibberellins + ↑ Ethylene	0.14 and 4.3	3 and 2
GA3 & STS	↑ Gibberellins + ↓ Ethylene	0.58 and 6	5 and 3

**Table 2:** Summary of pleiotropic effects of treatments. Compounds used in treatments include de-ionized water (control), gibberellic acid (GA3), paclobutrazol (PBZ), ethephon (ETH), silver thiosulfate (STS), methyl jasmonate (MeJ), diethyl dithiocarbamate (DIECA), indole acetic acid (IAA), phenoxyphenyl boronic acid (POBA), and 6-benzylamino purine (BAP).

Treatment	Pleiotropic Effects on Morphology
GA3	Larger and paler leaves, thicker stems, longer internode length
PBZ	Darker leaves, short internode length, reduced leaf senescence
ETH	Short internode length
STS	Signs of phytotoxicity at site of application
MeJ	Darker leaves, reduced leaf senescence
DIECA	Mottled leaf color, signs of phytotoxicity at site of application
IAA	Thick gnarled stems, reduced branching
POBA	Increased branching
BAP	Shoot tip necrosis

## Data Availability

The original contributions presented in this study are included in the article/Supplementary Materials. Further inquiries can be directed to the corresponding author.

## Supplementary Materials

The following supporting information can be downloaded at: https://www.mdpi.com/article/10.3390/plants15091291/s1. References [72,73,74,75,76,77] are cited in the supplementary materials.

## Abbreviations

The following abbreviations are used in this manuscript:

ABA	Abscisic acid
ADF	Age-dependent flower
BAP	6-benzylaminopurine
DIECA	Diethyl dithiocarbamate
DIECA and ETH	Diethyl dithiocarbamate and ethephon
DIECA and STS	Diethyl dithiocarbamate and silver thiosulfate
ETH	Ethephon
GA	Gibberellins
GA3	Gibberellic acid
GA3&STS	Gibberellic acid and silver thiosulfate
IAA	Indole acetic acid
MeJ	Methyl jasmonate
PBZ	Paclobutrazol
PBZ&ETH	Paclobutrazol and ethephon
PDI	Photoperiod-dependent inflorescence
POBA	Phenoxy phenylboronic acid
STS	Silver thiosulfate

## References

[1] Jiang H., Wang L., Merlin M.D., Clarke R.C., Pan Y., Zhang Y., Xiao G., Ding X. (2016). Ancient Cannabis Burial Shroud in a Central Eurasian Cemetery. Econ. Bot..

[2] Kobayashi M., Monohara A., Okitsu S., Yanagisawa S., Okamoto T. (2008). Fossil Hemp Fruits in the Earliest Jomon Period from the Okinoshima Site, Chiba Prefecture. Jpn. J. Hist. Bot..

[3] Long T., Wagner M., Demske D., Leipe C., Tarasov P.E. (2017). Cannabis in Eurasia: Origin of Human Use and Bronze Age Trans-Continental Connections. Veg. Hist. Archaeobot..

[4] Gurdev S. (1999). Green Revolution: Preparing for the 21st Century. Genome.

[5] Schluttenhofer C., Yuan L. (2017). Challenges towards Revitalizing Hemp: A Multifaceted Crop. Trends Plant Sci..

[6] Conneely L.J., Mauleon R., Mieog J., Barkla B.J., Kretzschmar T. (2021). Characterization of the *Cannabis sativa* Glandular Trichome Proteome. PLoS ONE.

[7] Faux A.M., Draye X., Lambert R., D’Andrimont R., Raulier P., Bertin P. (2013). The Relationship of Stem and Seed Yields to Flowering Phenology and Sex Expression in Monoecious Hemp (*Cannabis sativa* L.). Eur. J. Agron..

[8] Horkay E., Bócsa I. (1996). Objective Basis for Evaluation of Differences in Fibre Quality between Male, Female and Monoecious Hemp. J. Int. Hemp Assoc..

[9] Amaducci S., Zatta A., Pelatti F., Venturi G. (2008). Influence of Agronomic Factors on Yield and Quality of Hemp (*Cannabis sativa* L.) Fibre and Implication for an Innovative Production System. Field Crops Res..

[10] Chailakhyan M.K., Khryanin V.N. (1982). Hormonal Regulation of Sex Expression and Age-Related Changes. Sexuality in Plants and Its Hormonal Regulation.

[11] Spitzer-Rimon B., Duchin S., Bernstein N., Kamenetsky R. (2019). Architecture and Florogenesis in Female *Cannabis sativa* Plants. Front. Plant Sci..

[12] Eckels M., Heins R., Bugbee B. (2026). Unraveling the Confusion of Flowering Types and Terminology in *Cannabis sativa*. HortScience.

[13] Mishchenko S., Mokher J., Laiko I., Burbulis N., Kyrychenko H., Dudukova S. (2017). Phenological Growth Stages of Hemp (*Cannabis sativa* L.): Codification and Description According to the BBCH Scale. Žemės Ūkio Mokslai.

[14] Raman V., Lata H., Chandra S., Khan I.A., Elsohly M.A. (2017). Morpho-Anatomy of Marijuana (*Cannabis sativa* L.). Cannabis sativa L.—Botany and Biotechnology.

[15] Mediavilla V., Jonquera M., Schmid-Slembrouck I., Soldati A. (1998). Decimal Code for Growth Stages of Hemp (*Cannabis sativa* L.). J. Int. Hemp Assoc..

[16] Andre C.M., Hausman J.F., Guerriero G. (2016). *Cannabis sativa*: The Plant of the Thousand and One Molecules. Front. Plant Sci..

[17] Livingston S.J., Quilichini T.D., Booth J.K., Wong D.C.J., Rensing K.H., Laflamme-Yonkman J., Castellarin S.D., Bohlmann J., Page J.E., Samuels A.L. (2020). Cannabis Glandular Trichomes Alter Morphology and Metabolite Content during Flower Maturation. Plant J..

[18] Salentijn E.M.J., Petit J., Trindade L.M. (2019). The Complex Interactions between Flowering Behavior and Fiber Quality in Hemp. Front. Plant Sci..

[19] Dellaporta S.L., Calderon-urrea A. (1993). Sex Determination in Flowering Plants. Plant Cell.

[20] Ainsworth C. (2000). Boys and Girls Come out to Play: The Molecular Biology of Dioecious Plants. Ann. Bot..

[21] Punja Z.K., Holmes J.E. (2020). Hermaphroditism in Marijuana (*Cannabis sativa* L.) Inflorescences—Impact on Floral Morphology, Seed Formation, Progeny Sex Ratios, and Genetic Variation. Front. Plant Sci..

[22] Garcia-de Heer L., Mieog J., Burn A., Kretzschmar T. (2024). Why Not XY? Male Monoecious Sexual Phenotypes Challenge the Female Monoecious Paradigm in *Cannabis sativa* L.. Front. Plant Sci..

[23] Truta E., Surdu S., Zamfirache M.M., Oprica L. (2007). Some Aspects of Sex Determinism in Hemp. Analele Ştiinţifice Ale Univ. Alexandru Ioan Cuza Secţiunea Genet. Biol. Mol..

[24] Moliterni V.M.C., Cattivelli L., Ranalli P., Mandolino G. (2004). The Sexual Differentiation of *Cannabis sativa* L.: A Morphological and Molecular Study. Euphytica.

[25] Divashuk M.G., Alexandrov O.S., Razumova O.V., Kirov I.V., Karlov G.I. (2014). Molecular Cytogenetic Characterization of the Dioecious *Cannabis sativa* with an XY Chromosome Sex Determination System. PLoS ONE.

[26] Sakamoto K., Akiyama Y., Fukui K., Kamada H., Satoh S. (1998). Characterization; Genome Sizes and Morphology of Sex Chromosomes in Hemp (*Cannabis sativa* L.). Cytologia.

[27] Khryanin V.N. (2002). Role of Phytohormones in Sex Differentiation in Plants. Russ. J. Plant Physiol..

[28] Chandler J.W. (2011). The Hormonal Regulation of Flower Development. J. Plant Growth Regul..

[29] Pannell J.R. (2017). Plant Sex Determination. Curr. Biol..

[30] Galoch E. (1978). The Hormonal Control of Sex Differentiation in Dioecious Plants of Hemp (*Cannabis sativa*): The Influence of Plant Growth Regulators on Sex Expression in Male and Female Plants. Acta Soc. Bot. Pol..

[31] Adal A.M., Doshi K., Holbrook L., Mahmoud S.S. (2021). Comparative RNA-Seq Analysis Reveals Genes Associated with Masculinization in Female *Cannabis sativa*. Planta.

[32] Monthony A.S., Roy J., de Ronne M., Carlson O., Murch S.J., Torkamaneh D. (2026). Sex-specific Ethylene Responses Drive Floral Sexual Plasticity in *Cannabis sativa*. Plant J..

[33] Monthony A.S., de Ronne M., Torkamaneh D. (2024). Exploring Ethylene—Related Genes in *Cannabis sativa*: Implications for Sexual Plasticity. Plant Reprod..

[34] Garcia-de Heer L., Guo Q., Mieog J., Nolan M., Liu L., Dimopoulos N., Melzer R., Kretzschmar T. (2025). A Transcriptomic Analysis of Ethephon-Induced Sex Reversion of Male *Cannabis sativa* L. Reveals Changes in Expression of Floral Homeotic Genes and a Distinct Trichome Morphology. J. Exp. Bot..

[35] Chailakhyan M.K. (1979). Genetic and Hormonal Regulation of Growth, Flowering, and Sex Expression in Plants. Am. J. Bot..

[36] Durand B., Durand R. (1991). Sex Determination and Reproductive Organ Differentiation in Mercurialis. Plant Sci..

[37] Lubell J.D., Brand M.H. (2018). Foliar Sprays of Silver Thiosulfate Produce Male Flowers on Female Hemp Plants. Horttechnology.

[38] Flajšman M., Slapnik M., Murovec J. (2021). Production of Feminized Seeds of High CBD *Cannabis sativa* L. by Manipulation of Sex Expression and Its Application to Breeding. Front. Plant Sci..

[39] Garcia-de Heer L., Mieog J., Burn A., Nolan M., Liu L., Kretzschmar T., Liu B., Eunice Manansala S. (2026). Weeding out Variability: A Proof-of-Concept for Producing Uniform F1 Hybrid *Cannabis sativa* L. Using Single Seed Descent. Hortic. Res..

[40] Ram H.Y.M., Jaiswal V.S. (1970). Induction of Female Flowers on Male Plants of *Cannabis sativa* L. by 2-Chloroethanephos-Phonic Acid. Experientia.

[41] Chailakhyan M.K., Khryanin V.N. (1978). The Influence of Growth Regulators Absorbed by the Root on Sex Expression in Hemp Plants. Planta.

[42] Herich R. (1960). Gibberellin and Sex Differentiation of Flowering Plants. Nature.

[43] DiMatteo J., Kurtz L., Lubell-Brand J.D. (2020). Pollen Appearance and in Vitro Germination Varies for Five Strains of Female Hemp Masculinized Using Silver Thiosulfate. HortScience.

[44] Atal C.K. (1959). Sex Reversal in Hemp by Application of Gibberellin. Curr. Sci..

[45] Verma N., Kumar R., Kaur J. (2018). Maintenance of Gynoecious Lines of Cucumber through Modification of Sex Expression Using Gibberellic Acid, Silver Nitrate and Silver Thiosulphate in Cucumber (*Cucumis sativus* L.). Int. J. Curr. Microbiol. Appl. Sci..

[46] Thongplew P., Kangsopa J., Hermhuk S., Tongkoom K., Bhuyar P., Insalud N. (2025). Optimizing Ethephon Concentrations for Male Plant Feminization and Enhanced Seed Yield in Dioecious Thai Hemp (*Cannabis sativa* L. Cv. RPF3). Int. J. Plant Biol..

[47] Grabowska L., Mankowska G., Orlov N.N., Orlova L.G. (2004). Application of 2-Chloroethylphosphonic Acid in Breeding of Monoecious Hemp. J. Nat. Fibers.

[48] Sriram N., Ram M.Y.H. (1984). Sex-Associated Differences in Peroxidases and Ethylene Production and Their Modification by Ethephon Treatment in the Flowers of *Cannabis sativa* L.. Curr. Sci..

[49] Li Q., Guo W., Chen B., Pan F., Yang H., Zhou J., Wang G., Li X. (2021). Transcriptional and Hormonal Responses in Ethephon-Induced Promotion of Femaleness in Pumpkin. Front. Plant Sci..

[50] Ram M.H.Y., Jaiswal V.S. (1972). Induction of Male Flowers on Female Plants of *Cannabis sativa* by Gibberellins and Its Inhibition by Abscisic Acid. Planta.

[51] Dugardeyn J., Vandenbussche F., Van Der Straeten D. (2008). To Grow or Not to Grow: What Can We Learn on Ethylene-Gibberellin Cross-Talk by in Silico Gene Expression Analysis?. J. Exp. Bot..

[52] Weiss D., Ori N. (2007). Mechanisms of Cross Talk between Gibberellin and Other Hormones. Plant Physiol..

[53] Fang S., Duan Y., Nie L., Zhao W., Wang J., Zhao J., Zhao L., Wang L. (2022). Distinct Metabolic Profiling Is Correlated with Bisexual Flowers Formation Resulting from Exogenous Ethephon Induction in Melon (*Cucumis melo* L.). PeerJ.

[54] Hu Y., Vandenbussche F., Van Der Straeten D. (2017). Regulation of Seedling Growth by Ethylene and the Ethylene–Auxin Crosstalk. Planta.

[55] Spitzer-Rimon B., Shafran-Tomer H., Gottlieb G.H., Doron-Faigenboim A., Zemach H., Kamenetsky-Goldstein R., Flaishman M. (2022). Non-Photoperiodic Transition of Female Cannabis Seedlings from Juvenile to Adult Reproductive Stage. Plant Reprod..

[56] Song S., Qi T., Wasternack C., Xie D. (2014). Jasmonate Signaling and Crosstalk with Gibberellin and Ethylene. Curr. Opin. Plant Biol..

[57] Ferguson B.J., Beveridge C.A. (2009). Roles for Auxin, Cytokinin, and Strigolactone in Regulating Shoot Branching. Plant Physiol..

[58] Abdel-Moniem A. (2016). Effect of Some Growth Retardants on Growth and Flowering of *Helianthus annuus* L. Cv. Sunrich Orange Summer 981V Plants A—Effect of Foliar Spray Treatments with Ancymidol, Daminozide and Ethephon. Sci. J. Flowers Ornam. Plants.

[59] Obeso J.R. (2002). The Costs of Reproduction in Plants. New Phytol..

[60] Hazell P.B.R. (2009). The Asian Green Revolution.

[61] Orozco-Meléndez L.R., Hernández-Rodríguez O.A., Cruz-álvarez O., Robles-Hernández L., Ávila-Quezada G.D., Chavez E.S., Porras-Flores D.A., Ojeda-Barrios D.L. (2022). Paclobutrazol and Its Use in Fruit Production: A Review. Phyton-Int. J. Exp. Bot..

[62] Hedden P., Sponsel V. (2015). A Century of Gibberellin Research. J. Plant Growth Regul..

[63] Small E. (2018). Dwarf Germplasm: The Key to Giant Cannabis Hempseed and Cannabinoid Crops. Genet. Resour. Crop Evol..

[64] Hoppen C., Groth G. (2020). Novel Insights into the Transfer Routes of the Essential Copper Cofactor to the Ethylene Plant Hormone Receptor Family. Plant Signal. Behav..

[65] Faux A.M., Draye X., Flamand M.C., Occre A., Bertin P. (2016). Identification of QTLs for Sex Expression in Dioecious and Monoecious Hemp (*Cannabis sativa* L.). Euphytica.

[66] Bürkner P.C. (2017). Brms: An R Package for Bayesian Multilevel Models Using Stan. J. Stat. Softw..

[67] R Core Team (2022). R: A Language and Environment for Statistical Computing.

[68] Georgiou G.P. (2024). Bayesian Models Are More Sensitive than Frequentist Models in Identifying Differences in Small Datasets Comprising Phonetic Data. Stats.

[69] Jackson H., Shou Y., Azad N.A.B.M., Chua J.W., Perez R.L., Wang X., de Kraker M.E.A., Mo Y. (2025). A Comparison of Frequentist and Bayesian Approaches to the Personalised Randomised Controlled Trial (PRACTical)—Design and Analysis Considerations. BMC Med. Res. Methodol..

[70] Wickham H. (2016). Ggplot2: Elegant Graphics for Data Analysis.

[71] Ram M.H.Y., Jaiswal V.S. (1971). Feminization of Male Flowers of *Cannabis sativa* L. by a Morphactin. Naturwissenschaften.

[72] Ram M.H.Y., Sett R. (1979). Sex Reversal in the Female Plants of *Cannabis sativa* by Cobalt Ion. Proc. Indian Acad. Sci..

[73] Ram M.H.Y., Sett R. (1982). Induction of Fertile Male Flowers in Genetically Female *Cannabis sativa* Plants by Silver Nitrate and Silver Thiosulphate Anionic Complex. Theor. Appl. Genet..

[74] Soldatova N.A., Khryanin V.N. (2010). The Effects of Heavy Metal Salts on the Phytohormonal Status and Sex Expression in Marijuana. Russ. J. Plant Physiol..

[75] Kaushal S. (2012). Impact of Physical and Chemical Mutagens on Sex Expression in *Cannabis sativa*. Indian J. Fundam. Appl. Life Sci..

[76] Wizenberg S.B., Muir-Guarnaccia J., Campbell L.G. (2023). Cosexuality Reduces Pollen Production and Fitness in *Cannabis sativa* L.. Plants.

[77] Kim J., Kim D.G., Kim W.J., Lee Y.J., Lee S.H., Ryu J., Kim J.H., Kim S.H. (2024). Characterization of Male Flower Induction by Silver Thiosulfate Foliar Spray in Female Cannabis at the Middle Reproductive Stage for Breeding. Plants.

